# “Peripheral Neuropathy Crippling Bronchial Asthma”: Two Rare Case Reports of Churg-Strauss Syndrome

**DOI:** 10.1155/2014/673906

**Published:** 2014-12-16

**Authors:** Kamal Kishore Pandita, Khalid Javid Bhat, Sushil Razdan, R. P. Kudyar

**Affiliations:** Department of Internal Medicine, ASCOMS & Hospitals, Jammu, Jammu and Kashmir 180017, India

## Abstract

Churg-Strauss syndrome (CSS) is a rare cause of vasculitic neuropathy. Although rare and potentially fatal, Churg-Strauss syndrome (CSS) is easily diagnosable and treatable. The presence of bronchial asthma with peripheral neuropathy in a patient alerts a physician to this diagnosis. This is vividly illustrated by the presented two cases who had neuropathy associated with bronchial asthma, eosinophilia, sinusitis, and positive perinuclear antineutrophil cytoplasmic antibodies (p-ANCA) test, which improved with administration of steroids.

## 1. Introduction

The diagnosis of peripheral neuropathies can be challenging and time-consuming. Careful clinical and electrodiagnostic assessment, with attention to the pattern of nerve involvement, narrows the differential diagnosis and encourages a prompt treatment. Peripheral neuropathy is common in many vasculitic syndromes and can present as isolated nonsystemic vasculitic neuropathy (NSVN) or can be a part of systemic vasculitides like polyarteritis nodosa, systemic lupus erythematosus, and Churg-Strauss syndrome [1].

Churg-Strauss syndrome (CSS) is a systemic disorder characterized by asthma, hypereosinophilia, and systemic vasculitis and frequently involves peripheral nerves and skin. Untreated, CSS may be fatal and up to 50% die within three months of the onset of vasculitis but early treatment promises an excellent clinical response [2]. Discussed below are two patients of bronchial asthma with peripheral neuropathy due to CSS.

## 2. Case Report 1

**Figure 1:**
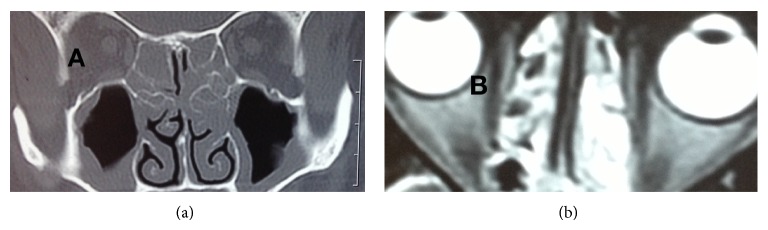
CT scan (a) and MRI scan (b) showing exuberant mucosal thickening of maxillary sinus and nose.

A 45-year-old ethnic Kashmiri woman presented in August 2004 with a few-weeks history of pain and discomfort in her lower limbs. For the past one week, she had low back pain radiating to left lower limb and weakness of the left foot. She also complained of distal paresthesias and pin prick-like feeling over her trunk. She had no bladder and bowel symptoms. She had history of chronic sinusitis and difficult-to-treat asthma with frequent exacerbations for the past several years. On examination, she had mild pallor and bilateral wheeze on chest auscultation. Her neurological examination revealed hypoesthesia along the right ulnar distribution and weak interossei of the right hand. Calf muscles of the left limb were wasted with weak dorsiflexion and planter flexion of the foot and diminished knee and ankle jerks on the same side. The rest of the systemic examination was unremarkable. Electrodiagnostic studies revealed severe left peroneal, tibial, and mild right ulnar neuropathies. These findings favoured the diagnosis of mononeuritis multiplex. She had hemoglobin level of 10.8 gm/dL, total leucocyte count (TLC) of 25000/mm^3^, differential count which revealed 20% eosinophils, and erythrocytic sedimentation rate (ESR) of 10 mm at first hour. Serum concentration of glucose, urea, creatinine, aminotransferases, thyrotropin, and electrolytes were within normal limits. Chest X-ray, electrocardiogram, and MRI of the lumbosacral spine were unremarkable. MRI of the head and CT scan of paranasal sinuses revealed pansinusitis with exuberant mucosal thickening and polyp formation (Figure 1). Perinuclear antineutrophil cytoplasmic antibody (p-ANCA) test was reactive at 34.2 U/mL (reference range < 7.0 U/mL) while tests for rheumatoid factor and antinuclear antibody (ANA) were negative. With these findings of asthma, sinusitis, mononeuritis multiplex, peripheral eosinophilia, and positive test for p-ANCA, we made the diagnosis of Churg-Strauss syndrome. We administered oral prednisolone for six weeks at a dose of 50 mg/day. She showed a remarkable recovery with minor sensory symptoms persisting. She was seen recently doing well on 5 mg alternate day prednisolone and azathioprine 100 mg daily.

## 3. Case Report 2

**Figure 2:**
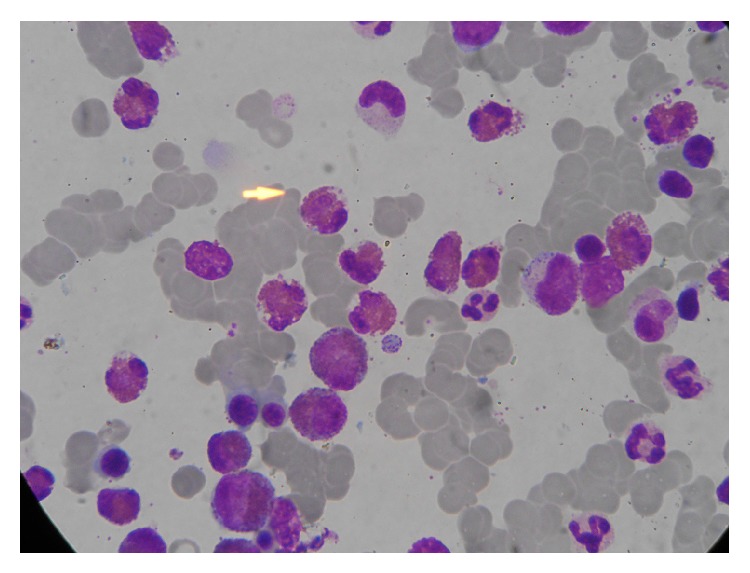
Bone marrow picture showing markedly increased number of eosinophils. (Leishman stain ×1000.)

A 40-year-old ethnic Kashmiri man presented in January 2009 with symptoms of burning paresthesias and motor weakness of the distal parts of his limbs, associated with feverish feel, over the past ten days. Symptoms first started with the left foot and progressively involved the hands, making it difficult to hold even a glass of water. Preceding the onset of neurological symptoms, he complained of nasal stuffiness, cough, wheezing, and breathlessness for three to four months. He had no history of urinary incontinence, toxin exposure, or substance abuse. He had bilateral wheeze on chest auscultation and spirometry showed a pattern consistent with bronchial asthma (FEV1: 2.87 L, FEV1/FVC: 68% predicted normal, and postbronchodilator FEV1: 3.24 L, 13% increment). Neurological examination revealed bilateral foot drop, symmetrical weakness of knee flexors, and asymmetrical weakness of the hand muscles (lower limbs > upper limbs). Ankle jerks were absent bilaterally. Touch and pain sensation was also diminished over right foot and palmar aspect of right hand. The rest of his general and systemic examination was unremarkable. He had hemoglobin level of 10 gm/dL and TLC of 16500/mm^3^ with differential count showing 50% eosinophils and absolute eosinophil count of 7000/mm^3^. Serum levels of urea, creatinine, bilirubin, and electrolytes were within normal limits. Urine examination revealed mild (2+) albuminuria. X-ray chest and tests for ANA, p-ANCA, and rheumatoid factor were normal. Electrodiagnostic studies revealed severe bilateral common peroneal, posterior tibial, right ulnar, and median neuropathies. In view of his asthma, eosinophilia, and peripheral neuropathy, Churg-Strauss syndrome was suspected and oral steroids were started. At follow-up, four weeks later he reported improvement in motor power and sensory symptoms and albuminuria resolved. His steroids were tapered off to an alternate day maintenance regimen and azathioprine 100 mg was started. He would get infrequent exacerbations of varying severity of his respiratory symptoms in 2010 and 2011 along with eosinophilia (>20%), which improved with short escalated courses of steroids. In October 2012, he again presented to the emergency department with three-weeks history of fever (101°F), recurrent vomiting, and body pains. He had a TLC of 21,800 cells/mm^3^ with differential count showing 46% eosinophils and absolute eosinophil count of 10,028/mm^3^ and ESR of 65 mm at the 1st hour. Urine examination revealed 3+ albuminuria. His urine and blood cultures were sterile. Although his general condition improved after hydration and intravenous antibiotics, his fever and vomiting improved dramatically only after administration of intravenous steroids. He tested positive for p-ANCA with bone marrow aspirate showing 40% eosinophils (Figure 2). When last seen in December 2012 he was doing well on low dose oral steroids and azathioprine daily.

## 4. Discussion

Identification of cause is one of the important goals of clinician's approach to a patient with newly diagnosed peripheral neuropathy. Evaluating a patient of neuropathy by thorough history taking, clinical examination, and relevant laboratory investigations for presence of any associated medical conditions, which may serve as a clue to the diagnosis, is an important tool to accomplish this goal. A structured approach based on a careful clinical and electrodiagnostic assessment with attention to the pattern of nerve involvement can help narrow the differential diagnosis and rationalise laboratory evaluation [3]. Systemic vasculitis should always be considered when a mononeuropathy multiplex is associated with systemic symptoms [4]. Our first patient had mononeuritis multiplex which is the second most common manifestation of CSS and occurs in 72% of these patients [5]. Common peroneal nerve is frequently involved, while ulnar, medial, and radial nerves are involved to a lesser degree in CSS [6]. Cranial nerve palsies are much rarer. In patient number 2, the pattern of symmetrical peripheral neuropathy was seen, which is less common and often develops in the absence of treatment. The two patients presented above had neuropathy associated with bronchial asthma and peripheral eosinophilia, which served as a clue to the diagnosis of CSS.

CSS, a systemic necrotizing vasculitis, is rare and although the annual incidence in general population is low (2.4 to 13 per million people), it is relatively high in asthma patients (34.6 to 64.6 per million persons) [2]. The asthma seen in CSS is notable for being of late onset and frequently severe and there is often no personal or family history of atopy [7]. No single clinical, laboratory, or histological feature is pathognomonic of the condition. Therefore, diagnostic criteria specified by American College of Rheumatology is commonly used and established when four of the criteria are met: (1) bronchial asthma, (2) eosinophilia (>1500/*μ*L or >10% of total leucocyte count), (3) migratory pulmonary infiltrates, (4) paranasal sinus abnormalities, (5) mononeuropathy or polyneuropathy, and (6) demonstration of extravascular infiltrates on tissue biopsy [8]. The pathophysiology may include direct endothelial injury by eosinophil derived neurotoxins, which may contribute to the development of peripheral neuropathy [9]. p-ANCA provides considerable value in supporting the diagnosis of clinically diagnosed CSS. Prognosis of untreated CSS is poor and guided by the revised five-factor score (FFS) of French vasculitis study which includes age >65 years, cardiac symptoms, renal dysfunction, gastrointestinal involvement, and absence of ear, nose, and throat involvement. The presence of one factor has a five-year mortality rate of 21%, and two or more factors indicate very severe disease with 40% five-year mortality rate [6]. However, treatment with corticosteroids and, when required, immunosuppressants, the outcome is dramatically good, with five-year survival exceeding 90% [10]. In both of our patients who had started with crippling peripheral neuropathies, early steroid use, along with steroid sparing azathioprine, improved the quality of life and reduced morbidity associated with the systemic vasculitis of CSS.

Although CSS is rare, clinicians can ill afford to misdiagnose it, since it is easily diagnosable and treatable and potentially fatal in the absence of treatment. The phrase “asthmatic neuropathy” may serve as a ready reminder to connote the common underlying pathophysiology of apparently two different clinical entities.
